# Environmentally relevant concentration of caffeine—effect on activity and circadian rhythm in wild perch

**DOI:** 10.1007/s11356-022-19583-3

**Published:** 2022-03-17

**Authors:** Daniel Cerveny, Petr Cisar, Tomas Brodin, Erin S. McCallum, Jerker Fick

**Affiliations:** 1 Department of Wildlife, Fish and Environmental Studies, Swedish University of Agricultural Sciences, SE-90183 Umeå, Sweden; 2 Faculty of Fisheries and Protection of Waters, South Bohemian Research Center of Aquaculture and Biodiversity of Hydrocenoses, University of South Bohemia in Ceske Budejovice, Zátiší 728/II, 389 25, Vodňany, Czech Republic; 3 Department of Chemistry, Umeå University, SE-90187 Umeå, Sweden

**Keywords:** Coffee, Exposure, Fish, Behavior, Swimming, Mass spectrometry

## Abstract

We studied the ecological consequences of widespread caffeine contamination by conducting an experiment focused on changes in the behavioral traits of wild perch (*Perca fluviatilis*) after waterborne exposure to 10 μg L^−1^ of caffeine. We monitored fish swimming performance during both light and dark conditions to study the effect of caffeine on fish activity and circadian rhythm, using a novel three-dimensional tracking system that enabled positioning even in complete darkness. All individuals underwent three behavioral trials—before exposure, after 24 h of exposure, and after 5 days of exposure. We did not observe any effect of the given caffeine concentration on fish activity under light or dark conditions. Regardless of caffeine exposure, fish swimming performance was significantly affected by both the light-dark conditions and repeating of behavioral trials. Individuals in both treatments swam significantly more during the light condition and their activity increased with time as follows: before exposure < after 24 h of exposure < after 5 days of exposure. We confirmed that the three-dimensional automated tracking system based on infrared sensors was highly effective for conducting behavioral experiments under completely dark conditions.

**Supplementary Information:**

The online version contains supplementary material available at 10.1007/s11356-022-19583-3.

## Introduction

Pharmaceuticals and personal care products (PPCPs) have become contaminants of great interest over the last few decades because of their increasing production and use by a growing human population. A wide range of PPCPs, including illicit drugs, food-additives, and plastic by-products, in concentrations from ng L^−1^ to μg L^−1^ has been reported by numerous studies in surface water and groundwater bodies from both freshwater and marine aquatic environments (aus der Beek et al. 2016, Biel-Maeso et al. 2018, Charuaud et al. 2019, Kasprzyk-Hordern et al. 2008, Meffe & de Bustamante 2014, Sui et al. 2015). Most of those PPCPs enter the aquatic environments via urban sewage treatment plant (STP) effluents, because conventional technologies used at STPs are not capable of completely removing such a wide range of PPCPs (Azzouz & Ballesteros 2013, Baker &Kasprzyk-Hordern 2013, Lindberg et al. 2014, Yang et al. 2017). Although concentrations of PCPPs occurring in the environment are not acutely toxic, they may still exert sub-lethal effects in aquatic organisms, e.g., altering fish metabolism (Burkina et al. 2015, Du et al. 2018), inducing endocrine disruption (Niemuth et al. 2015), or affecting early life development (Zhang et al. 2015). Specific adverse effects have been expected with exposure to psychoactive pharmaceuticals, as several authors have reported that exposure alters natural fish behavior (Brodin et al. 2013, Kellner et al. 2016, McCallum et al. 2017).

Caffeine (1,3,7-trimethylxanthine) is an alkaloid that naturally occurs in many plant species. With its global daily consumption of estimated to be 460 tons (Moore et al. 2008), caffeine is the most commonly used stimulant by humans. It is object of recreational use through a various types of beverages (coffee, tea, soft drinks, energy drinks) or food products (chocolate, desserts) (Barone & Roberts 1996, Reyes & Cornelis 2018). Occurrence of caffeine in the aquatic environments has been reported worldwide (Cantwell et al. 2016, Comeau et al. 2008, Cui et al. 2019, Ide et al. 2017, Rodríguez-Gil et al. 2018, Zhou et al. 2010) with concentrations up to 1000 μg L^−1^ in Costa Rica (Spongberg et al. 2011). Nevertheless, concentrations in surface waters typically lay below 1 μg L^−1^, with a maximum concentration of 39.8 μg L^−1^ in Europe reported by Zhou et al. (2019a). As measurable concentrations of caffeine are being found in surface waters globally and because of the direct linkage between its occurrence and human activity, caffeine was proposed to be a suitable marker of anthropogenic contamination in aquatic environments (Buerge et al. 2003, Ferreira et al. 2005, Metcalfe et al. 2003).

Humans use caffeine because it functions to stimulate the nervous system. Specifically, caffeine acts as an adenosine antagonist, which means that it interferes with adenosine receptors (primarily A1 and A2a receptors). Caffeine therefore affects sleep-wake regulation and psychomotor functions because adenosine plays an important role in these processes (Einöther & Giesbrecht 2013). However, effects on the nervous system are still much more complex in humans and caffeine has been found to affect attention, alertness, fatigue, reaction time, or accuracy in a variety of tasks (Einöther & Giesbrecht 2013). Some of those effects are also dose dependent, as locomotor activity was reported to increase following low to moderate dose, but it decreased with higher doses (Marin et al. 2011). As the A1 and A2a adenosine receptors were evolutionary conserved across the vertebrate taxa including fish (Boehmler et al. 2009), similar behavioral alterations might be expected in non-target organisms due to caffeine presence in the aquatic environments.

Despite its reported presence in the aquatic environments worldwide, not many studies have yet been performed to assess for any adverse effects of caffeine in non-target organisms. Aguirre-Martínez et al. (2015) reported effects of caffeine on biomarkers of phases I and II metabolism in marine bivalve mollusk species (*Corbicula fluminea*) at concentrations as low as 0.1 μg L^−1^. An increased level of lipid peroxidation, an indicator of oxidative stress, was reported for two Polychaeta species after 28 days exposure to caffeine at concentration of 0.5 μg L^−1^ (Pires et al. 2016). Very limited information is available regarding the possible effect of caffeine on important ecological endpoints, such as its potential to alter fish behavior. Zhou et al. (2019a) reported an effect on zebrafish (*Danio rerio*) larvae (5 days post fertilization) locomotor activity at concentrations of 1, 10, and 100 μg L^−1^, and these effects were only recorded during the dark regime of the behavioral assay. Zebrafish larvae were used as an experimental species by Steele et al. (2018), who registered the effect of caffeine on locomotor activity at environmentally relevant concentrations. Other studies have been performed to study behavioral effects of caffeine on fish (Faillace et al. 2018, Ladu et al. 2015, Neri et al. 2019, Ruiz-Oliveira et al. 2019), but those were using concentrations over 10000 μg L^−1^, and thus, such conditions are far from those occurring in real environments. It is also worth noting that all the abovementioned studies focusing on the behavioral endpoints of caffeine were conducted using laboratory-bred individuals and there is currently no information about effects on wild fish.

The daily rhythms are generally very important for all living organisms and are being expressed on all levels of biological organization starting with gene expression and cell mitosis at the individual level and scaling up to the behavior of whole communities within given ecosystem (Patiño et al. 2011, Tamai et al. 2012). Daily and seasonal light cycles represent the most important factor that keeps the organism’s circadian clock synchronized with the environment and allow an organism to thrive in its natural habitat (Pando & Sassone-Corsi 2002). While the circadian rhythm of physiology processes can be considered relatively stable as it is directly controlled by circadian clocks that regulate the melatonin secretion based on the light regime (Sánchez-Vázquez et al. 2019), the behavior might more likely be disrupted by multiple environmental stressors, including neuroactive chemicals.

The aim of present study was to evaluate possible effect of an environmentally relevant concentration of caffeine on the swimming performance and circadian rhythm of juvenile European perch (*Perca fluviatilis*) originating from a wild population. Based on the documented mode of action and effects reported for humans (Einöther & Giesbrecht 2013), we hypothesized that exposure to caffeine will lead to (1) increased activity and (2) disruption of circadian rhythm in perch.

## Material and methods

### Experimental fish

We chose European perch (*Perca fluviatilis*) as a model organism because it represents a common freshwater fish species in Europe. Using a beach seine, we collected young-of-the-year perch at the shoreline of Lake Bjännsjön (Umeå Municipality, Sweden) in June 2018. Fish were transported to an aerated, flow-through tank at the Umeå University, where they were kept until experiment. The tank was continuously fed by non-chlorinated tap water and fish were fed daily with frozen chironomid larvae (feeding rate approximately 3% of fish biomass). During first 4 weeks of acclimatization period, the dark/light regime was slowly shifted every day to reach 12/12 h ratio with light from midnight until noon to facilitate experimental logistics. The experimental animals were handled in accordance with Ethical Committee on Animal Experiments in Umeå (dnr: A18-15), current Swedish law, and institutional guidelines for the protection of human subjects and animal welfare (European parliament and Council, 2010).

### Caffeine exposure

As the caffeine half-life in water was reported to be approximately 1.5 days (Lam et al. 2004), a semi-static exposure scenario with daily water renewal was chosen. Fish (*n*=64) were exposed to either a control (0 μg L^−1^) or caffeine (10 μg L^−1^) treatment in individual 6L plastic (polypropylene) containers resulting in 32 individuals per treatment. The criterion for selected concentration was its environmental relevance as reported caffeine concentrations in European surface waters range from <LOQ to 39.8 μg L^−1^ (Zhou et al. 2019b). Fish were transferred from the holding tank into individual exposure containers 3 days before the first behavioral trial to acclimate, and no caffeine was administered at this point. The experiment took 8 days in total (3 days of acclimatization + 5 days of exposure), and each fish underwent behavioral trials and three time points: before the exposure, 24 h post-exposure, and 5 days post-exposure. Fish were exposed in a temperature-controlled room to keep the same stable temperature and the same light-dark regime during the exposure as in the holding tanks. During the experiment, fish were fed daily with frozen chironomid larvae. The water in the experimental containers was renewed (100%) every day in both treatments using aged tap water of the same physical-chemical properties as in holding tank. In case of caffeine treatment, water was spiked after renewal with caffeine using a stock solution of 2 mg L^−1^. Water of randomly selected tanks from both treatments was sampled both before and after water renewal to assess caffeine exposure and possible contamination of control tanks.

### Behavioral trial design

**Fig. 1:**
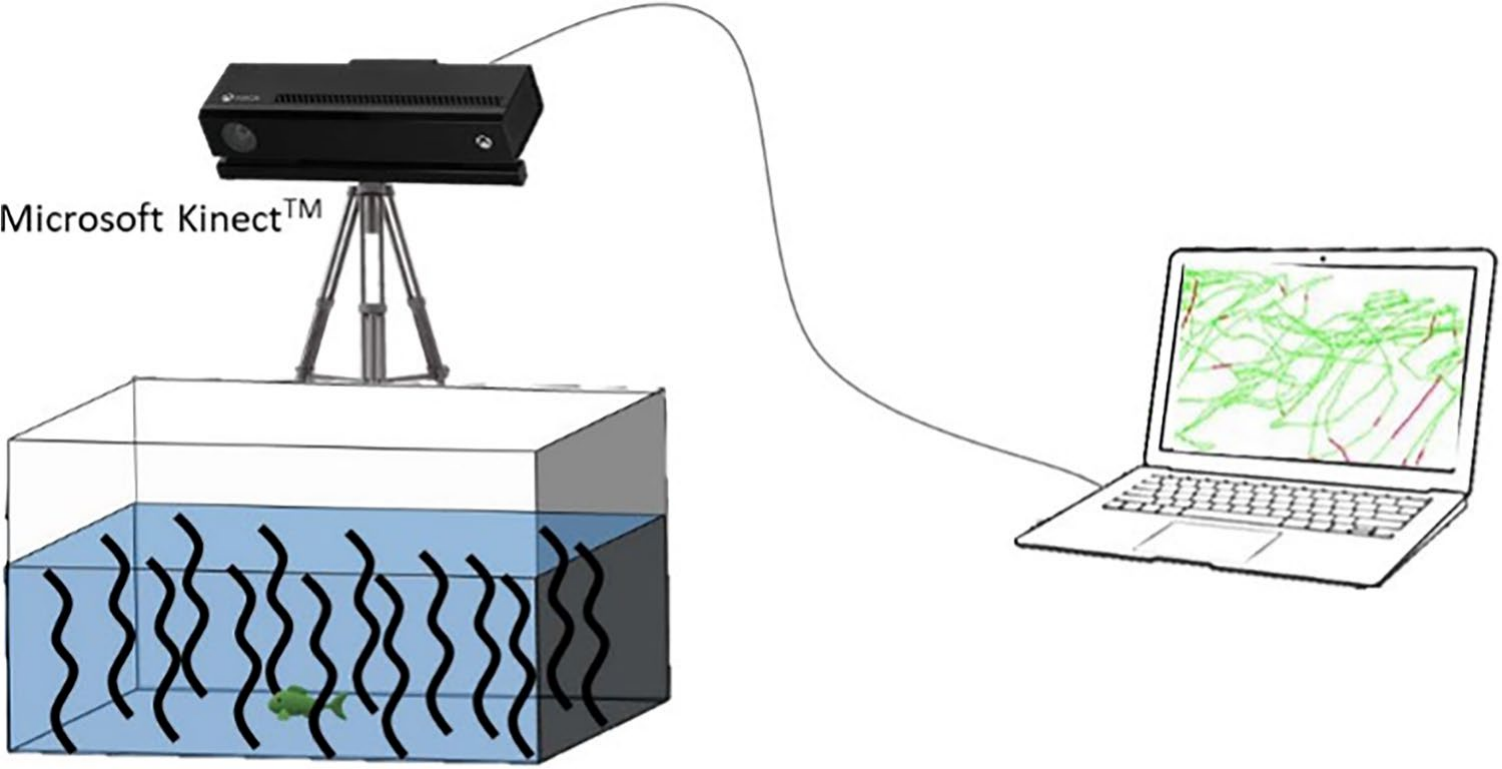
Diagram of the activity behavioral trial experimental aquarium, showing the positioning of the tracking technology above the tank

As stated above, each fish underwent behavioral trials at three time points in both the light and dark regime. At each time point, activity during the light regime was first assessed, followed by the same test during the dark regime. Fish were allowed to rest for 75 min in their individual containers between the light and dark regime trials. As all individuals underwent behavioral trials at three time points, the whole experiment was done in a staggered design, where fish were divided into eight groups (each containing four individuals of each treatment). This was mainly due to the fact that one temperature-controlled room was used for both the exposure and the behavioral trials, and there was a limited number of tracking devices meaning that we could only do four individuals at the same time. Detailed information about the experiment schedule is provided in Supplementary material (Table S1). During the trial, each perch was introduced individually into the center of a glass behavioral testing aquaria (60×34 cm) with water depth of 25 cm and contained a plastic artificial vegetation to provide shelter and reduce perch anxiety behavior (Fig. 1). The behavioral testing aquaria were filled with water from a fishless flow through holding tank every day before the trials started. When testing behavior of fish from the caffeine treatment, the water in aquaria was spiked with caffeine to reach the same nominal concentration as in exposure containers. After being introduced in testing aquarium, the fish was left undisturbed and its movements were tracked for 75 min. The first 15 min were excluded when processing the data representing a period for fish to acclimatize. Activity was quantified as the total distance swam during the 60 min of tracking. Immediately after their last behavior testing, each individual fish was euthanized by overdose with tricaine methanesulfonate (MS 222) at concentration of 0.3 mg L^−1^, placed in the labeled polyethylene bag, and sored at −18 °C until samples extraction and analysis.

### Automated tracking system

Behavioral trials were recorded with a monitoring system using the Microsoft Kinect^TM^ Sensor V2. The sensor consists of both an emitter and receiver of a specific wavelength of infrared light and it is able to capture the three-dimensional position of moving fish under any light conditions. Positions are recorded into the log file with a sampling frequency of 30 records per second and a spatial resolution of 2 mm (Saberioon & Cisar 2016). Converting these positions into 3D fish tracks was done by an in-house implemented software. Functionality of the tracking system for the normal light conditions was evaluated in our previous work (Cerveny et al. 2020).

### Chemicals and reagents

For the purpose of sample preparation and their instrumental analysis, LC/MS grade of acetonitrile and methanol (LiChrosolv—hypergrade) were purchased from Merck (Darmstadt, Germany). Formic acid (Sigma-Aldrich, Steinheim, Germany) was used to prepare the 0.1% mobile phases used in liquid chromatography and mass spectrometry. Caffeine (CAS 58-08-2) and isotopically labeled caffeine ^13^C_3_ (CAS 78072-66-9) were obtained from Sigma-Aldrich. Working mixtures of standards were prepared in methanol at a concentration of 100 ng mL^−1^ and stored at −18 °C. Ultra-pure water was prepared by a Milli-Q Gradient water system (Millipore, Billerica, USA).

### Preparation of samples for chemical analysis

Samples of water were defrosted at room temperature. Then, 5 mL was passed through a 0.45 μm syringe filter Filtropur S (Sarstedt, Nümbrecht, Germany) into 10 mL autosampler glass vials, and 5 ng of internal standard was added. For the tissue samples, fish were defrosted at room temperature, and then 0.1 g of muscle tissue from dorsal part of the body and whole brain were sampled into 2 mL polypropylene tubes from each fish. To extract caffeine from tissue samples, 1.5 mL of acetonitrile was added to the tubes together with 10 zirconium beads and samples were spiked with 50 ng of internal standard. Homogenization at 42000 oscillations per minute (Mini Beadbeater, BioSpec Bartlesville, USA) and centrifugation at 17500*g* for 10 min (Beckman Coulter Microfuge 22R Centrifuge) followed. The supernatant was pipetted into the 12-mL glass vial and whole extraction process was repeated with new 1.5 mL acetonitrile. The supernatants from both extractions were combined, evaporated to near dryness (TurboVap Classic LV, Biotage AB, Sweden), the eluent was reconstituted with 150 μL of methanol, and transferred into the 2 mL autosampler vials equipped with 200 μL inserts. The final extracts were frozen for a minimum of 24 h to ensure protein precipitation and centrifuged again directly before analysis.

### Instrumental analysis

Water analysis followed the protocol of Fick et al. (2017) based on an online solid-phase extraction system coupled with liquid chromatography-tandem mass spectrometry (SPE LC-MS/MS), which has been also described in previous work (Khan et al. 2012). All water and tissue extract samples were analyzed using a triple stage quadrupole mass spectrometer (TSQ Quantiva,Thermo Scientific, San Jose, CA) equipped with a heated-electrospray ionization (HESI) ion source. The instrument was coupled to an Accela LC pump (Thermo Fisher Scientific, San Jose, CA) and a PAL HTC autosampler (CTC Analytics AG, Zwingen, Switzerland). Target analytes were separated using a C18 phase Hypersil gold column (50 mm × 2.1 mm ID × 3 μm particles, Thermo Fisher Scientific, San Jose, CA, USA) before tandem mass spectrometry analysis. In addition to these described above, the liquid chromatography for water analysis also included a Surveyor LC-Pump (Thermo Fisher Scientific, San Jose, CA, USA) and an on-line SPE Hypersil GOLD C18 column (20 mm × 2.1 mm ID × 12 μm particles, Thermo Fisher Scientific, Waltham, MA, USA).

The analytical method was evaluated regarding its linearity, limit of quantification (LOQ), and recovery. LOQ was derived from a nine-point calibration curve with concentrations ranging from 1 to 10000 ng L^−1^ (water samples) and from a seven-point calibration curve with concentrations ranging from 0.1 to 50 ng g^−1^ (tissue samples). Peak area corresponding to the lowest point for which the method was linear (r^2^ > 0.999) was then used for calculation of LOQs in individual samples. Corresponding LOQ values reflect differences among IS recovery between samples and ranged from 10 to 40 ng L^−1^ for water samples and from 2.8 to 13 ng g^−1^ for samples of fish tissues. An internal standard approach was used for quantification of caffeine in both water and fish tissue samples.

To assess recovery, 16 fortified samples (eight for each sampled tissue, brain, and muscle) were prepared by adding 5 ng sample^−1^ of caffeine native standard before the extraction procedure. The mean recovery of 106% and 70% was achieved for fish muscle and brain tissue, respectively.

Several blank samples, including the tap water, Milli-Q water, and mobile phase solvents, were analyzed. Quantifiable concentrations of caffeine were not detected in blank samples, but some contamination corresponding to low ng L^−1^ was present. For this reason, the peak area measured in Milli-Q water blank sample was subtracted from all water samples and likewise the peak area detected in the methanol solvent blank was subtracted from all fish tissue samples.

### Statistical analysis

After testing the dataset for normality (Shapiro-Wilk and Kolmogorov-Smirnov tests), nonparametric Kruskal-Wallis ANOVA tests were performed to evaluate differences in activity between the treatments and light regimes for each time point (i.e., before the exposure, 24 hours post-exposure, and 5 days post-exposure). The overall Kruskal-Wallis tests were followed by post hoc multiple comparisons of mean ranks (two-sided significance levels with a Bonferroni adjustment). Statistical analyses of differences between the treatments and light regimes were done using Statistica 12 software (StatSoft Inc., USA).

Friedman tests and Wilcoxon signed ranks post hoc comparisons were used to analyze the difference in activity between the time points at which behavior trials were run. Friedman and Wilcoxon tests were conducted with R (version: 4.1.0; R Core Team 2021) using the package “rstatix” (Kassambara 2021).

## Results and discussion

### Chemical analyses

In total, 83 water samples were analyzed. Twenty of these represented control treatments (either from exposure tanks or behavioral testing aquaria). The others were sampled from caffeine treatment tanks at different time points—before and after the water renewal or from testing arenas used for behavioral trials that were run after the exposure to caffeine. Unfortunately, caffeine concentrations above the LOQ were found in all measured samples of water originated from control treatments. This contamination could be caused by aeration of exposure tanks producing microscopic droplets and their subsequent transport between the tanks, as both control and caffeine treatment occupied the same temperature-controlled room. In 2 of 12 samples taken from the control treatment exposure tanks, caffeine concentration exceeded 100 ng L^−1^, while it was below 50 ng L^−1^ in most of the rest of analyzed. However, this still represents a concentration of caffeine that is two to three orders of magnitude lower in the control than in the exposure treatments.

Concerning the caffeine treatment group, no difference in concentration was registered for samples taken before and after the water renewal, indicating low degradation of caffeine. This finding is in contrast with the work of Lam et al. (2004) who found that the half-life of caffeine in water was approximately 1.5 days. However, their experiment took place in microcosm design and they proposed UV irradiation as a main factor of caffeine degradation, which might explain the difference in our study that was done in laboratory conditions. Mean water concentrations measured in both exposure tanks and behavior arenas are summarized in Table 1, and all individual values are presented in Supplementary material (Table S2).

**Table 1:** Average caffeine concentrations measured in exposure tanks and behavior arenas

Treatment	Caffeine concentration in μg L^−1^ , mean ± SD (number of samples)		
	Testing arena	Exposure tank fresh^1^	Exposure tank 24H^1^
Control	0.09 ± 0.051 (*N*=8)	NA	0.08 ± 0.064 (*N*=12)
Exposed	9.03 ± 1.777 (*N*=15)	9.23 ± 1.802 (*N*=24)	9.34 ± 1.815 (*N*=24)

^1^To evaluate caffeine stability, water was sampled after (fresh) and before (24h) the regular daily water renewal in exposure tanks allocated to caffeine treatment. Control tanks were sampled only after 24 h (before the water renewal)

To analyze the concentration of caffeine in fish tissues, a subsample of 18 fish from each treatment was sampled, resulting in 72 samples (muscle and brain) being analyzed. No quantifiable concentrations of caffeine were found in samples originated from control fish, while the concentrations quantified in tissues of fish exposed to 10 μg L^−1^ of caffeine ranged from <LOQ (one individual brain sample) to 68 ng g^−1^. No statistically significant difference was observed between caffeine concentrations measured in the brain and in the muscle of exposed fish. The slightly lower concentrations measured in fish brains are likely the result of lower recovery of caffeine observed for this tissue in fortified samples rather than difference in tissue-specific bioconcentration. Concentrations of caffeine measured in tissue samples are presented in Table 2 together with the morphological characteristics of the experimental fish. All measured individual tissue concentrations are given in Supplementary material (Table S3). The fish assigned to control and caffeine exposure treatments did not differ in total body length or body weight.

**Table 2:** Morphological characteristics of the experimental fish and results of chemical analyses; mean ± SD

Treatment	*N*	Length (cm)	Weight (g)	Measured caffeine concentration	
				Muscle (ng g^−1^)	Brain (ng g^−1^)
Control treatment	18	8.7 ± 1.0	4.9 ± 1.8	<LOQ	<LOQ
Caffeine exposure	18	8.5 ± 0.7	4.3 ± 1.1	42 ± 10.5	36 ± 9.4

### Fish behavior analysis

**Fig. 2:**
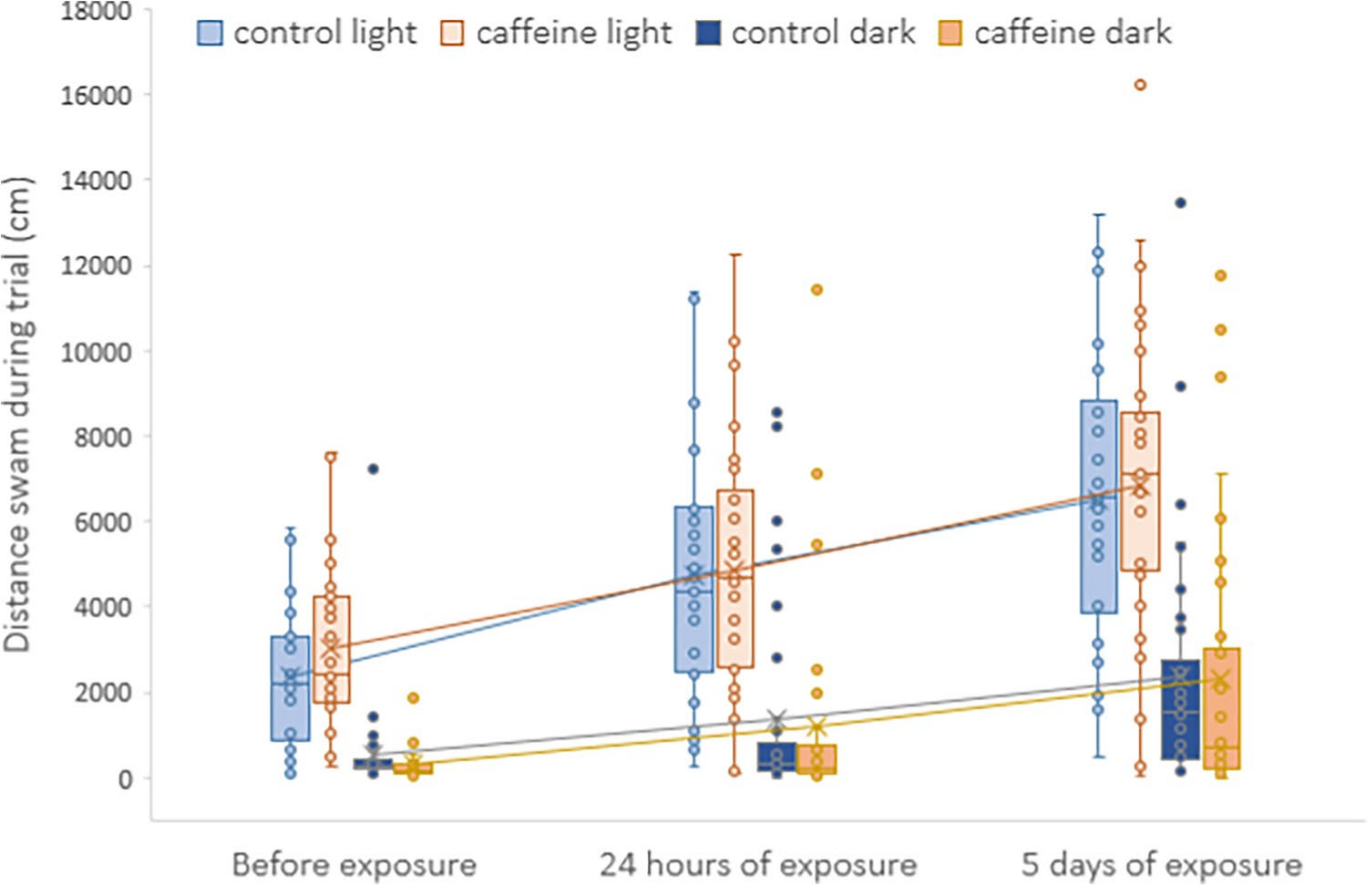
Activity of perch, defined as the total distance swam (cm) during 60 min behavioral trial that each fish underwent before exposure, 24 h after exposure, and after 5 days of exposure to either control or caffeine (10 μ L^−1^) treatment. Raw data (circles), upper and lower quartiles (boxes) with variability outside the quartiles (whiskers), and average values (crosses connected with lines) for both light and dark conditions during behavior trials are presented

Regardless of the light conditions during the trial, no significant differences in activity of perch were found between the control and caffeine treatments at any of three time points of the repeated behavioral trials (before exposure, after 24 h of exposure, after 5 days of exposure). Our results do not show an effect of tested caffeine exposure on perch activity or circadian rhythm during the given experimental laboratory conditions (Fig. 2, Table S4).

Limited information to date is available on the effects of caffeine at environmentally relevant concentrations on fish. Zhou et al. (2019a) reported an effect of caffeine on zebrafish locomotor activity at 1 μg L^−1^. Similar results were found by Steele et al. (2018) for zebrafish, but not for fathead minnow (*Pimephales promelas*). Both of these studies were performed on laboratory reared fish larvae that were exposed to caffeine before organogenesis was completed and the behavioral trials were run up to 118 h post fertilization. At this stage, there is likely low biotransformation capacity related to limited cytochrome P450 1 (CYP1) activity in immature liver of zebrafish larvae (Alderton et al. 2010, Saad et al. 2016). As the enzymes of CYP family are known to be responsible for detoxification processes, it might be assumed that their lower activity results in higher bioconcentration and subsequently increased effect of various xenobiotics. However, this is only one suggested explanation for why no effect of caffeine was observed in our study and more factors such as species-specific sensitivity or difference between laboratory reared and wild fish can also play important roles. Also, we cannot exclude the possibility that the behavior of our control fish was affected by the contamination of control treatments stated above. Nevertheless, there was at least two orders of magnitude difference in the caffeine water concentration between the control and exposed fish and still no difference in activity was registered. Moreover, we did not detect quantifiable levels of caffeine in the tissues of control fish.

We noted significant differences (Supplementary material, Tables S4, S5) in perch activity induced by both the light regime and the time point of the behavioral trial (Fig. 2). Concerning the circadian rhythm of perch, all fish from both treatments and across all time points swam more during the light conditions. Such behavior is typical for this species and so this finding is in agreement with previously published works performed in natural habitats (Hölker et al. 2007, Zamora & Moreno-Amich 2002). The time point of the behavioral trials presented another important factor that significantly affects activity of perch in our experiment. Fish from both treatments and both light conditions increased their activity during the behavioral trials as followed: before exposure < after 24 h of exposure < after 5 days of exposure (Fig. 2). We suggest that the increase in fish activity is related to a decrease of stress induced by conducting the behavioral trials repeatedly in a separate testing arena: in the first trial, the testing arena is novel and becomes more familiar with each successive trial. Also, the fish may habituate to being kept in individual exposure containers, which could manifest increased exploration or activity in across the behavioral trials. A similar shift in behavior was registered in our previous experiment with benzodiazepines where the boldness of wild perch increased with time that the individuals were kept in laboratory conditions (Cerveny et al. 2020).

## Conclusions

This is the first study evaluating the potential of caffeine to induce behavioral changes in wild fish at an environmentally relevant concentration. Neither activity nor circadian rhythms were affected in perch after exposure to 10 μg L^−1^ of caffeine in our experiment. However, there are more behavioral effects resulting from caffeine consumption reported in humans (e.g., attention, alertness, and reaction time) that were not part of this study. More research is thus necessary to evaluate responsibly the effects of this widespread anthropogenic contaminant on fish behavior in a variety of contexts. Regardless of caffeine treatment, both light regime and time point of behavioral trials affected perch activity. Finally, we confirm that this automated tracking system based on infrared technology is very effective at tracking behavior under completely dark conditions.

## Supplementary information

ESM 1 (PDF 224 kb)

## References

[CR1] Aguirre-Martínez GV, DelValls AT, Laura Martín-Díaz M (2015). Yes, caffeine, ibuprofen, carbamazepine, novobiocin and tamoxifen have an effect on Corbicula fluminea (Müller, 1774). Ecotoxicology and Environmental Safety.

[CR2] Alderton W, Berghmans S, Butler P, Chassaing H, Fleming A, Golder Z, Richards F, Gardner I (2010). Accumulation and metabolism of drugs and CYP probe substrates in zebrafish larvae. Xenobiotica.

[CR3] aus der Beek T, Weber FA, Bergmann A, Hickmann S, Ebert I, Hein A, Küster A (2016). Pharmaceuticals in the environment-Global occurrences and perspectives. Environmental Toxicology and Chemistry.

[CR4] Azzouz A, Ballesteros E (2013). Influence of seasonal climate differences on the pharmaceutical, hormone and personal care product removal efficiency of a drinking water treatment plant. Chemosphere.

[CR5] Baker DR, Kasprzyk-Hordern B (2013). Spatial and temporal occurrence of pharmaceuticals and illicit drugs in the aqueous environment and during wastewater treatment: new developments. Science of the Total Environment.

[CR6] Barone JJ, Roberts HR (1996). Caffeine consumption. Food and Chemical Toxicology.

[CR7] Biel-Maeso M, Baena-Nogueras RM, Corada-Fernández C, Lara-Martín PA (2018). Occurrence, distribution and environmental risk of pharmaceutically active compounds (PhACs) in coastal and ocean waters from the Gulf of Cadiz (SW Spain). Science of the Total Environment.

[CR8] Boehmler W, Petko J, Woll M, Frey C, Thisse B, Thisse C, Canfield VA, Levenson R (2009). Identification of zebrafish A2 adenosine receptors and expression in developing embryos. Gene Expr Patterns.

[CR9] Brodin T, Fick J, Jonsson M, Klaminder J (2013). Dilute concentrations of a psychiatric drug alter behavior of fish from natural populations. Science.

[CR10] Buerge IJ, Poiger T, Müller MD, Buser HR (2003). Caffeine, an anthropogenic marker for wastewater contamination of surface waters. Environmental Science and Technology.

[CR11] Burkina V, Zlabek V, Zamaratskaia G (2015). Effects of pharmaceuticals present in aquatic environment on Phase I metabolism in fish. Environmental Toxicology and Pharmacology.

[CR12] Cantwell MG, Katz DR, Sullivan JC, Borci T, Chen RF (2016). Caffeine in Boston Harbor past and present, assessing its utility as a tracer of wastewater contamination in an urban estuary. Marine Pollution Bulletin.

[CR13] Cerveny D, Brodin T, Cisar P, McCallum ES, Fick J (2020) Bioconcentration and behavioral effects of four benzodiazepines and their environmentally relevant mixture in wild fish. Science of the Total Environment 702. 10.1016/j.scitotenv.2019.134780 10.1016/j.scitotenv.2019.134780 31733557

[CR14] Charuaud L, Jarde E, Jaffrezic A, Thomas MF, Le Bot B (2019). Veterinary pharmaceutical residues from natural water to tap water: sales, occurrence and fate. Journal of Hazardous Materials.

[CR15] Comeau F, Surette C, Brun GL, Losier R (2008). The occurrence of acidic drugs and caffeine in sewage effluents and receiving waters from three coastal watersheds in Atlantic Canada. Science of the Total Environment.

[CR16] Cui Y, Wang Y, Pan C, Li R, Xue R, Guo J, Zhang R (2019). Spatiotemporal distributions, source apportionment and potential risks of 15 pharmaceuticals and personal care products (PPCPs) in Qinzhou Bay, South China. Marine Pollution Bulletin.

[CR17] Du SNN, McCallum ES, Vaseghi-Shanjani M, Choi JA, Warriner TR, Balshine S, Scott GR (2018). Metabolic costs of exposure to wastewater effluent lead to compensatory adjustments in respiratory physiology in bluegill sunfish. Environmental Science and Technology.

[CR18] Einöther SJL, Giesbrecht T (2013). Caffeine as an attention enhancer: Reviewing existing assumptions. Psychopharmacology.

[CR19] Faillace MP, Pisera-Fuster A, Bernabeu R (2018). Evaluation of the rewarding properties of nicotine and caffeine by implementation of a five-choice conditioned place preference task in zebrafish. Progress in Neuro-Psychopharmacology and Biological Psychiatry.

[CR20] Ferreira AP, De Lourdes C, Da Cunha N (2005). Anthropic pollution in aquatic environment: development of a caffeine indicator. International Journal of Environmental Health Research.

[CR21] Fick J, Brodin T, Heynen M, Klaminder J, Jonsson M, Grabicova K, Randak T, Grabic R, Kodes V, Slobodnik J, Sweetman A, Earnshaw M, Caracciolo AB, Lettieri T, Loos R (2017). Screening of benzodiazepines in thirty European rivers. Chemosphere.

[CR22] Hölker F, Dörner H, Schulze T, Haertel-Borer SS, Peacor SD, Mehner T (2007). Species-specific responses of planktivorous fish to the introduction of a new piscivore: implications for prey fitness. Freshwater Biology.

[CR23] Ide AH, Osawa RA, Marcante LO, da Costa PJ, de Azevedo JCR (2017) Occurrence of pharmaceutical products, female sex hormones and caffeine in a subtropical region in Brazil. Clean - Soil, Air, Water 45. 10.1002/clen.201700334

[CR24] Kasprzyk-Hordern B, Dinsdale RM, Guwy AJ (2008). The occurrence of pharmaceuticals, personal care products, endocrine disruptors and illicit drugs in surface water in South Wales, UK. Water Research.

[CR25] Kellner M, Porseryd T, Hallgren S, Porsch-Hällström I, Hansen SH, Olsén KH (2016). Waterborne citalopram has anxiolytic effects and increases locomotor activity in the three-spine stickleback (Gasterosteus aculeatus). Aquatic Toxicology.

[CR26] Khan GA, Lindberg R, Grabic R, Fick J (2012). The development and application of a system for simultaneously determining anti-infectives and nasal decongestants using on-line solid-phase extraction and liquid chromatography-tandem mass spectrometry. Journal of Pharmaceutical and Biomedical Analysis.

[CR27] Ladu F, Mwaffo V, Li J, Macrì S, Porfiri M (2015). Acute caffeine administration affects zebrafish response to a robotic stimulus. Behavioural Brain Research.

[CR28] Lam MW, Young CJ, Brain RA, Johnson DJ, Hanson ML, Wilson CJ, Richards SM, Solomon KR, Mabury SA (2004). Aquatic persistence of eight pharmaceuticals in a microcosm study. Environmental Toxicology and Chemistry.

[CR29] Lindberg RH, Ostman M, Olofsson U, Grabic R, Fick J (2014). Occurrence and behaviour of 105 active pharmaceutical ingredients in sewage waters of a municipal sewer collection system. Water Research.

[CR30] Marin MF, Lord C, Andrews J, Juster RP, Sindi S, Arsenault-Lapierre G, Fiocco AJ, Lupien SJ (2011). Chronic stress, cognitive functioning and mental health. Neurobiology of Learning and Memory.

[CR31] McCallum ES, Bose APH, Warriner TR, Balshine S (2017). An evaluation of behavioural endpoints: the pharmaceutical pollutant fluoxetine decreases aggression across multiple contexts in round goby (Neogobius melanostomus). Chemosphere.

[CR32] Meffe R, de Bustamante I (2014). Emerging organic contaminants in surface water and groundwater: a first overview of the situation in Italy. Science of the Total Environment.

[CR33] Metcalfe CD, Miao XS, Koenig BG, Struger J (2003). Distribution of acidic and neutral drugs in surface waters near sewage treatment plants in the lower Great Lakes, Canada. Environmental Toxicology and Chemistry.

[CR34] Moore MT, Greenway SL, Farris JL, Guerra B (2008). Assessing caffeine as an emerging environmental concern using conventional approaches. Archives of Environmental Contamination and Toxicology.

[CR35] Neri D, Ruberto T, Mwaffo V, Bartolini T, Porfiri M (2019). Social environment modulates anxiogenic effects of caffeine in zebrafish. Behavioural Pharmacology.

[CR36] Niemuth NJ, Jordan R, Crago J, Blanksma C, Johnson R, Klaper RD (2015). Metformin exposure at environmentally relevant concentrations causes potential endocrine disruption in adult male fish. Environmental Toxicology and Chemistry.

[CR37] Pando MP, Sassone-Corsi P (2002). Unraveling the mechanisms of the vertebrate circadian clock: zebrafish may light the way. BioEssays.

[CR38] Patiño MAL, Rodríguez-Illamola A, Conde-Sieira M, Soengas JL, Míguez JM (2011). Daily rhythmic expression patterns of clock1a, bmal1, and per1 genes in retina and hypothalamus of the rainbow trout, Oncorhynchus mykiss. Chronobiology International.

[CR39] Pires A, Almeida Â, Calisto V, Schneider RJ, Esteves VI, Wrona FJ, Soares AMVM, Figueira E, Freitas R (2016). Long-term exposure of polychaetes to caffeine: biochemical alterations induced in Diopatra neapolitana and Arenicola marina. Environmental Pollution.

[CR40] Reyes CM, Cornelis MC (2018) Caffeine in the diet: country-level consumption and guidelines. Nutrients 10. 10.3390/nu10111772 10.3390/nu10111772 PMC6266969 30445721

[CR41] Rodríguez-Gil JL, Cáceres N, Dafouz R, Valcárcel Y (2018). Caffeine and paraxanthine in aquatic systems: global exposure distributions and probabilistic risk assessment. Science of the Total Environment.

[CR42] Ruiz-Oliveira J, Silva PF, Luchiari AC (2019) Coffee time: low caffeine dose promotes attention and focus in zebrafish. Learning and Behavior. 10.3758/s13420-018-0369-3 10.3758/s13420-018-0369-3 30623296

[CR43] Saad M, Verbueken E, Pype C, Casteleyn C, Van Ginneken C, Maes L, Cos P, Van Cruchten S (2016). In vitro CYP1A activity in the zebrafish: temporal but low metabolite levels during organogenesis and lack of gender differences in the adult stage. Reproductive Toxicology.

[CR44] Saberioon MM, Cisar P (2016). Automated multiple fish tracking in three-dimension using a structured light sensor. Computers and Electronics in Agriculture.

[CR45] Sánchez-Vázquez FJ, López-Olmeda JF, Vera LM, Migaud H, López-Patiño MA, Míguez JM (2019) Environmental cycles, melatonin, and circadian control of stress response in fish. Frontiers in Endocrinology 10. 10.3389/fendo.2019.00279 10.3389/fendo.2019.00279 PMC6579845 31244768

[CR46] Spongberg AL, Witter JD, Acuña J, Vargas J, Murillo M, Umaña G, Gómez E, Perez G (2011). Reconnaissance of selected PPCP compounds in Costa Rican surface waters. Water Research.

[CR47] Steele WB, Mole RA, Brooks BW (2018) Experimental protocol for examining behavioral response profiles in larval fish: application to the neuro-stimulant caffeine. Journal of Visualized Experiments 2018. 10.3791/57938 10.3791/57938 PMC6126542 30102268

[CR48] Sui Q, Cao X, Lu S, Zhao W, Qiu Z, Yu G (2015). Occurrence, sources and fate of pharmaceuticals and personal care products in the groundwater: a review. Emerging Contaminants.

[CR49] Tamai TK, Young LC, Cox CA, Whitmore D (2012). Light acts on the zebrafish circadian clock to suppress rhythmic mitosis and cell proliferation. Journal of Biological Rhythms.

[CR50] Yang Y, Ok YS, Kim K-H, Kwon EE, Tsang YF (2017). Occurrences and removal of pharmaceuticals and personal care products (PPCPs) in drinking water and water/sewage treatment plants: a review. Science of the Total Environment.

[CR51] Zamora L, Moreno-Amich R (2002). Quantifying the activity and movement of perch in a temperate lake by integrating acoustic telemetry and a geographic information system. Hydrobiologia.

[CR52] Zhang Q, Cheng J, Xin Q (2015). Effects of tetracycline on developmental toxicity and molecular responses in zebrafish (Danio rerio) embryos. Ecotoxicology.

[CR53] Zhou H, Wu C, Huang X, Gao M, Wen X, Tsuno H, Tanaka H (2010). Occurrence of selected pharmaceuticals and caffeine in sewage treatment plants and receiving rivers in Beijing, China. Water Environment Research.

[CR54] Zhou S, Chen Q, Di Paolo C, Shao Y, Hollert H, Seiler T-B (2019). Behavioral profile alterations in zebrafish larvae exposed to environmentally relevant concentrations of eight priority pharmaceuticals. Sci Total Environ.

[CR55] Zhou SB, Di Paolo C, Wu X, Shao Y, Seiler TB, Hollert H (2019). Optimization of screening-level risk assessment and priority selection of emerging pollutants - the case of pharmaceuticals in European surface waters. Environment International.

