# Supplementary material for: Environmentally relevant concentration of caffeine—effect on activity and circadian rhythm in wild perch
Source: Environ Sci Pollut Res Int. 2022 Mar 17;29(36):54264–72. doi: 10.1007/s11356-022-19583-3 (PMC9356920; doi:10.1007/s11356-022-19583-3)
Supplement: Supplementary file 1 — (PDF 224 kb) [file 11356_2022_19583_MOESM1_ESM.pdf]

Environmentally relevant concentration of caffeine - effect on activity and circadian rhythm in wild perch

Cerveny, D. [a, b*], Cisar, P.[b], Brodin, T.[a], McCallum, E.S[a]., Fick, J.[c]

*Corresponding author, cerveny@frov.jcu.cz

[a] Department of Wildlife, Fish and Environmental Studies, Swedish University of Agricultural Sciences, SE-90183, Umeå, Sweden

[b] University of South Bohemia in Ceske Budejovice, Faculty of Fisheries and Protection of Waters, South Bohemian Research Center of Aquaculture and Biodiversity of Hydrocenoses, Zátiší 728/II, 389 25 Vodňany, Czech Republic

[c] Department of Chemistry, Umeå University, SE-90187, Umeå, Sweden

Supplementary material

Table S1. Time schedule of the experiment with experimental data from behavior trials

group	fish ID	treatment	start[1]	behavior trials (dates, scores in cm of total swam distance)								
				before exposure			24 hours exposure			5 days exposure		
				date	score light	score dark	date	score light	score dark	date	score light	score dark
1	1	control	21.11.2018	24.11.2018	5580	823	25.11.2018	6429	4012	29.11.2018	6896	1167
	2				657	62		880	116		1597	638
	3				4367	291		8769	1435		9593	2165
	4				374	289		1039	229		5891	198
	5	caffeine			2031	55		2629	135		241	481
	6				1664	150		153	0		271	127
	7				5699	86		5547	379		11967	562
	8				4245	251		2548	672		1375	817
2	NA[2]	control	23.11.2018	26.11.2018			27.11.2018			1.12.2018		
	NA[2]											
	11				2627	383		3730	587		5453	2501
	12				1961	104		643	133		8573	109
	NA[2]	caffeine										
	NA[2]											
	15				5042	481		7345	1174		16247	795
	16				1882	150		5530	232		8459	202
3	17	control	29.11.2018	2.12.2018	5715	84	3.12.2018	4024	456	7.12.2018	6537	1964
	18				3269	271		5654	8218		7105	1232
	19				849	356		2588	583		2684	1495
	20				1790	104		8927	1108		2822	4418
	21	caffeine			3961	151		7448	5478		5264	4549
	22				4246	554		4244	421		4860	6071
	23				4491	103		108	119		69	125
	24				7479	120		12242	50		12571	10
4	25	control	1.12.2018	4.12.2018	3294	7231	5.12.2018	4923	607	9.12.2018	8137	9186
	26				2619	977		3850	321		5341	6373
	27				2148	326		5439	148		5165	333
	28				2519	286		1111	192		3997	747
	29	caffeine			1625	323		4740	84		8577	540
	30				2877	131		3451	7143		6244	7123
	31				2521	190		9654	545		5045	3307
	32				7594	82		3674	142		6273	154

[1] date when fish were moved into the individual containers, followed by three days of acclimatization before the first behavior trial and subsequent exposure

[2] four individuals (2 from each treatment) excluded from analysis due to the power failure at the building during behavior trial

Table S1, continued. Time schedule of the experiment with experimental data from behavior trials

group	fish ID	treatment	start[1]	before exposure: date	before exposure: score light	before exposure: score dark	24 hours exposure: date	24 hours exposure: score light	24 hours exposure: score dark	5 days exposure: date	5 days exposure: score light	5 days exposure: score dark
5	33	control	7.12.2018	10.12.2018	205	422	11.12.2018	2937	281	15.11.2018	9567	13470
	34				1012	577		3709	8544		13209	1745
	35				2106	261		2408	177		3372	5534
	36				5837	744		11197	555		12293	141
	37	caffeine			3729	505		8203	11419		10589	10467
	38				1063	195		2675	92		4027	87
	39				2338	140		8424	59		7169	220
	40				524	145		3271	134		8198	322
6	41	control	9.12.2018	12.12.2018	2280	473	13.12.2018	6480	5987	17.12.2018	11889	1621
	42				125	404		6004	743		1900	610
	43				1126	212		5362	0		6604	470
	44				124	142		2401	132		8274	162
	45	caffeine			1266	87		7209	186		6654	704
	46				243	71		4373	111		7826	187
	47				1714	119		4579	55		10933	586
	48				2115	365		1365	192		7135	2933
7	49	control	15.12.2018	18.12.2018	2132	196	19.12.2018	4660	99	23.12.2018	9718	550
	50				4385	267		11391	214		2099	3468
	51				3297	239		7676	93		6267	1946
	52				3863	247		3812	175		7466	300
	53	caffeine			5592	212		6071	1990		10003	901
	54				4239	834		1846	2550		7091	5095
	55				505	97		2087	82		6715	135
	56				2310	501		1344	865		8078	1429
8	57	control	17.12.2018	20.12.2018	716	1419	21.12.2018	285	2822	25.12.2018	5550	1742
	58				3055	155		6272	215		10132	216
	59				2434	287		7774	721		9583	3763
	60				768	113		4353	237		7606	1680
	61	caffeine			1810	110		1959	183		3256	253
	62				1971	67		6536	167		2816	660
	63				3311	130		5446	133		8257	1515
	64				2720	299		10208	259		8955	2099

Behavior trials: dates, scores in cm of total swam distance.

[1] date when fish were moved into the individual containers, followed by three days of acclimatization before the first behavior trial and subsequent exposure

Table S2. Measured concentrations (μg L^{-1}) of caffeine in randomly selected exposure tanks and behavior arenas.

		measured caffeine concentration	
Tank ID	Treatment	Fresh	24H
1	control		0.18
1	control		0.06
9	control		0.05
9	control		0.06
17	control		0.02
25	control		0.09
33	control		0.06
33	control		0.26
41	control		0.06
41	control		0.04
49	control		0.06
57	control		0.02
5	caffeine	12.02	12.15
5	caffeine	9.24	10.48
13	caffeine	7.86	10.81
13	caffeine	8.95	11.94
29	caffeine	10.58	7.02
29	caffeine	11.32	11.17
29	caffeine	10.90	10.96
37	caffeine	8.15	7.67
37	caffeine	7.88	6.71
37	caffeine	10.03	8.67
13	caffeine	10.13	6.74
21	caffeine	11.49	8.91
21	caffeine	11.93	9.99
21	caffeine	8.76	7.61
45	caffeine	7.22	8.42
45	caffeine	9.11	7.71
45	caffeine	6.88	8.37
53	caffeine	6.48	6.20
53	caffeine	9.42	11.74
53	caffeine	7.67	9.36
61	caffeine	8.37	10.06
61	caffeine	10.08	10.08
61	caffeine	5.62	10.07
61	caffeine	11.44	11.36
arena	control		0.15
arena	control		0.15
arena	control		0.04
arena	control		0.05

Table S2, continued. Measured concentrations ($\mu g\ L^{-1}$) of caffeine in randomly selected exposure tanks and behavior arenas.

		measured caffeine concentration	
Tank ID	Treatment	Fresh	24H
arena	control		0.15
arena	control		0.05
arena	control		0.04
arena	control		0.08
arena	caffeine		9.36
arena	caffeine		7.11
arena	caffeine		6.79
arena	caffeine		9.94
arena	caffeine		5.95
arena	caffeine		8.32
arena	caffeine		7.95
arena	caffeine		8.56
arena	caffeine		8.08
arena	caffeine		12.61
arena	caffeine		10.85
arena	caffeine		9.06
arena	caffeine		11.20
arena	caffeine		10.06
arena	caffeine		9.60

Table S3. Measured concentrations ($ng\ g^{-1}$) of caffeine in tissues of 18 control and 18 exposed individuals of fish subsampled for chemical analysis

Fish ID	Treatment	Muscle	Brain
1	control	<LOQ	<LOQ
2	control	<LOQ	<LOQ
3	control	<LOQ	<LOQ
4	control	<LOQ	<LOQ
9	control	<LOQ	<LOQ
10	control	<LOQ	<LOQ
11	control	<LOQ	<LOQ
12	control	<LOQ	<LOQ
17	control	<LOQ	<LOQ
18	control	<LOQ	<LOQ
19	control	<LOQ	<LOQ
20	control	<LOQ	<LOQ
25	control	<LOQ	<LOQ
26	control	<LOQ	<LOQ
27	control	<LOQ	<LOQ
28	control	<LOQ	<LOQ

Table S3, continued. Measured concentrations (ng g^{-1}) of caffeine in tissues of 18 control and 18 exposed individuals of fish subsampled for chemical analysis

Fish ID	Treatment	Muscle	Brain
33	control	<LOQ	<LOQ
34	control	<LOQ	<LOQ
5	caffeine	30.6	32.0
6	caffeine	40.5	39.7
7	caffeine	29.9	28.8
8	caffeine	34.4	31.3
13	caffeine	37.5	37.3
14	caffeine	40.9	41.1
15	caffeine	31.9	37.9
16	caffeine	58.0	42.5
21	caffeine	50.4	40.3
22	caffeine	39.2	34.4
23	caffeine	52.5	29.7
24	caffeine	68.1	47.6
29	caffeine	40.6	48.7
30	caffeine	31.7	38.4
31	caffeine	42.7	43.5
32	caffeine	54.4	40.5
37	caffeine	33.8	28.5
38	caffeine	32.9	32.6

Table S4. Results of statistical analysis—differences in fish activity between treatments at each of the time points when behavioral trials were run.

	Kruskal-Wallis ANOVA by ranks				Post-hoc comparisons[1], p-values			
	H-value	df	N	p-value		control light	caffeine light	control dark
Before exposure	69.9	3	120	<0.0001	caffeine light	1.0000		
					control dark	<0.0001	<0.0001	
					caffeine dark	<0.0001	<0.0001	0.5287
24 hours post exposure	50.284	3	120	<0.0001	caffeine light	1.0000		
					control dark	<0.0001	<0.0001	
					caffeine dark	<0.0001	<0.0001	1.0000
5 days post exposure	48.906	3	120	<0.0001	caffeine light	1.0000		
					control dark	<0.0001	0.00013	
					caffeine dark	<0.0001	<0.0001	1.0000

[1] Multiple comparisons of mean ranks, two-sided significance levels with a Bonferroni adjustment

Table S5. Results of statistical analysis—differences in fish activity between different time points of behavioral trials for each treatment (i.e. before exposure, after 24 hours of exposure, and after 5 days of exposure).

	Friedman ANOVA					Post-hoc comparisons	
	Chi Sqr.	df	N	p-value	Kendall´s W	Comparison	p-value
control light	38.4	2	30	<0.0001	0.64	Before vs. 24 hrs	<0.0001
						Before vs. 5 days	<0.0001
						24 hrs vs. 5 days	0.002
caffeine light	17.07	2	30	0.0002	0.26	Before vs. 24 hrs	0.005
						Before vs. 5 days	<0.0001
						24 hrs vs. 5 days	0.008
control dark	19.4	2	30	<0.0001	0.30	Before vs. 24 hrs	0.50
						Before vs. 5 days	<0.0001
						24 hrs vs. 5 days	0.13
caffeine dark	20.9	2	30	<0.0001	0.33	Before vs. 24 hrs	0.10
						Before vs. 5 days	<0.0001
						24 hrs vs. 5 days	0.017

[1] Multiple comparisons of mean ranks (Wilcoxon signed-ranks), two-sided significance levels with a Bonferroni adjustment
